# Interpopulational Variation in Cyclotide Production in Heavy-Metal-Treated Pseudometallophyte (*Viola tricolor* L.)

**DOI:** 10.3390/plants14030471

**Published:** 2025-02-05

**Authors:** Rebecca Miszczak, Blazej Slazak, Klaudia Sychta, Ulf Göransson, Anna Nilsson, Aneta Słomka

**Affiliations:** 1 Doctoral School of Exact and Natural Sciences, Jagiellonian University in Kraków, 11 Prof. S. Łojasiewicza St., 30-348 Kraków, Poland; rebecca.miszczak@doctoral.uj.edu.pl; 2 Department of Plant Cytology and Embryology, Institute of Botany, Faculty of Biology, Jagiellonian University in Kraków, 9 Gronostajowa St., 30-387 Kraków, Poland; klaudia.sychta@uj.edu.pl; 3 W. Szafer Institute of Botany, Polish Academy of Science, 46 Lubicz St., 31-512 Kraków, Poland; b.slazak@botany.pl; 4 Pharmacognosy, Department of Pharmaceutical Biosciences, Uppsala University, P.O. Box 591, 751 23 Uppsala, Sweden; ulf.goransson@uu.se; 5 Spatial Mass Spectrometry, Science for Life Laboratory, Department of Pharmaceutical Biosciences, Uppsala University, P.O. Box 591, 751 24 Uppsala, Sweden; anna.m.nilsson@uu.se

**Keywords:** antimicrobial peptides, MALDI-MS, mechanism of tolerance, zinc and lead

## Abstract

It remains an open question whether violets use universal mechanisms, such as the production of metallothioneins, phytochelatins, and organic acids and/or rely on specific mechanisms like the production of antimicrobial cyclic peptides (cyclotides) for heavy metal tolerance. To contribute to the understanding of the role of cyclotides, we used seed-derived plants from metallicolous (M) and non-metallicolous (NM) populations of *Viola tricolor*, a pseudometallophyte tolerant to Zn and Pb. Eight- to ten-week-old plants were treated with 1000 μM of Zn or Pb for 3 or 7 days and subsequently measured for cyclotides and heavy metal content using MALDI-MS and Atomic Absorption Spectrometry (AAS), respectively. Individuals from the M population accumulated a similar amount of Zn but occasionally more Pb in comparison with the NM population. Of the 18 different cyclotides included in the analysis, some showed statistically significant changes under the heavy metal treatment. In general, a decrease was observed in the M population, whereas an increase was observed in the NM population (except for the 3-day treatment with Zn). The day of treatment and dose of metal and their interaction played a crucial role in the explained variance for cyclotides produced by the M individuals but not for the NM plants. This unravels the importance of this antimicrobial compound in heavy metal tolerance and indicates that, in *V. tricolor*, cyclotides are involved in heavy metal tolerance, but specimens from two populations have developed different strategies and tolerance mechanisms involving cyclotides to mitigate heavy metal stress.

## 1. Introduction

The genus *Viola* (violets) is represented by many species tolerant to heavy metals (HMs), the so-called obligate metallophytes (occurring exclusively on heavy-metal-polluted sites) or pseudometallophytes (occurring at metalliferous and non-metalliferous sites). Their morphological variability, genetic diversity, and some physiological aspects of in situ adaptation to HMs have been described [1,2,3,4,5,6]. However, the tolerance mechanisms that render these plants above-average tolerance to metals are not clear. It has only been shown that in response to cadmium, the hyperaccumulator *Viola boashanensis* increases the expression of genes related to sucrose metabolism and tonoplast transporters [7]. Previous studies have also shown the involvement of universal mechanisms of HM tolerance, such as the production of glutathione, some phytochelatins, and organic acids in some metal-tolerant violets. Based on comparisons of violets with nontolerant and tolerant species from other genera, it was shown that tolerance to HMs, perhaps common to all species of the genus, was most likely inherited from a common ancestor(s), so called constitutive (innate) tolerance [6]. HM tolerance is present in both types of species/populations (metallicolous—M and non-metallicolous—NM), at organismal and cellular levels as shown by experimental studies involving growing non-metallophytes on metal-enriched soil, phytotoxicity tests, and the application of metals to hydroponic/cell culture [4,5,8,9,10]. Some authors have postulated that mycorrhizal fungi, involving specific fungal strains adapted to elevated concentrations of HMs in the soil, could enable violets to colonize metal-bearing areas [11,12,13].

In *V. tricolor*, a widely distributed Eurasian species classified as a pseudometallophyte, both M and NM populations occurring in many parts of the range could be Zn- and Pb-tolerant [2,12]. Experimental treatment with high concentrations of these metals (1000 μM and 100 μM, respectively) in hydroponic culture confirmed that even a high concentration of 1000 μM did not negatively affect chlorophyll fluorescence. It did not raise the levels of H_2_O_2_, indicating a lack of oxidative stress, which could be an indicator of HM stress [8].

An increasing number of studies have shown that compounds collectively called antimicrobial peptides (AMPs), including lipid transfer proteins, defensin, defensin-like proteins, and cyclotides, play a role in response to various kinds of stresses [14,15]. It remains unclear if they also play a role in HM detoxification. Few studies have shown that they may indeed be involved in the mechanism of HM tolerance. As demonstrated using genetic engineering methods, the defensins from the *pdf1* genes in the metallophyte *Arabidopsis halleri* promote tolerance to zinc levels in both yeast and *A. thaliana* (normally sensitive to the elevated levels of this element) [16,17]. Unlike metallothioneins and phytochelatins, defensins do not detoxify HMs in the vacuole but in the endoplasmic reticulum, where they decrease stress caused by the excess of Zn ions [18]. Therefore, it seems that different HM tolerance mechanisms may be used in different compartments of the plant cell. Some defensins, e.g., AtPDF2.6, can be released to the xylem vessel and bind Cd in the parenchyma xylem cells, preventing its long-distance transport as demonstrated in *Arabidopsis* [19]. Research on the role of AMPs in heavy metal tolerance is sparse so far; thus, it is not possible to draw broader conclusions and include them among the compounds responsible for metal detoxification.

The *Violaceae* family is one of the six families whose members constitutively produce dozens of thousands cyclotides belonging to AMPs [20]. These cyclic peptides, built from 27 to 37 amino acids stabilized by three disulfide bridges (cysteine knot motif), are known for their involvement in defense mechanisms against microorganisms and pests [21,22].

As for the AMP group as a whole, little is known about the role of cyclotides in abiotic stress, including heavy metals. This has only been an issue under consideration for several years. While it is known that -SH-group-rich compounds (e.g., phytochelatins, glutathione, and metalothioneins) are involved in HMs chelating and various membrane transporters located in the plasma membrane and tonoplast are responsible for their accumulation, whether these biotic stress-acting compounds may also be involved in defense against HMs has not yet been confirmed. Experiments with the use of extracted cyclotides have shown that they tend to bind to the metal and form an antibacterial biofilm on this metal [23]. Some studies also have shown that divalent cation coordination (including some HMs) is the invariant property of all cyclotides. The backbone amides of residues Thr 16, Cys 22, Asn 23, and Thr 24 and the side-chain nitrogen atoms of Asn 8 and Asn 23 are indicated as specific metal binding sites in isolated kalata B1 treated with Mn [24,25]. These few mentioned studies on the cyclotide–heavy metal context come, however, from ex vivo analyses. The only results on the biological role of cyclotides in HM tolerance are provided by a study by Sychta et al. [26], who showed these compounds co-localize with Pb (both deposited in vacuoles), suggesting that they have an affinity to bind this metal in the cells of *V. tricolor* treated with Pb.

Hence, the biological role of cyclotides in metal tolerance is therefore still unresolved. The goal of this study was (1) to test the ability of genetically divergent *V. tricolor* specimens (with a different life history associated with heavy metal exposure) to accumulate Zn and Pb administered under experimental conditions and (2) to indicate whether putative metal uptake under experimental conditions is associated with quantitative and qualitative changes in the production of cyclotides, compounds with high affinity for HMs. We conjectured that, since cyclotides could bind HMs, the amount of and variation in these compounds may vary depending on the metal type/dose/time of treatment. We also expected that the Mpopulation, being more tolerant to HMs, will also make stronger use of cyclotides (by being more responsive) to mitigate HM stress.

## 2. Results

### 2.1. Similar Accumulation of Zn and Pb in M and NM Specimens of Viola tricolor

The differences in Zn and Pb accumulation as a function of treatment time and population were insignificant (*p* > 0.05), except in the 3-day treatment with Pb on M plants. The specimens of both populations accumulated more Zn than Pb. A bioaccumulation factor (the concentration of metal within plants to the concentration of metal in the medium) for all samples under Zn treatment was well above 1, indicating a high potential for Zn accumulation (Table 1).

### 2.2. Variation in Cyclotide Production Between M and NM Individuals of Viola tricolor Under Treatment with Heavy Metals

**Figure 1:**
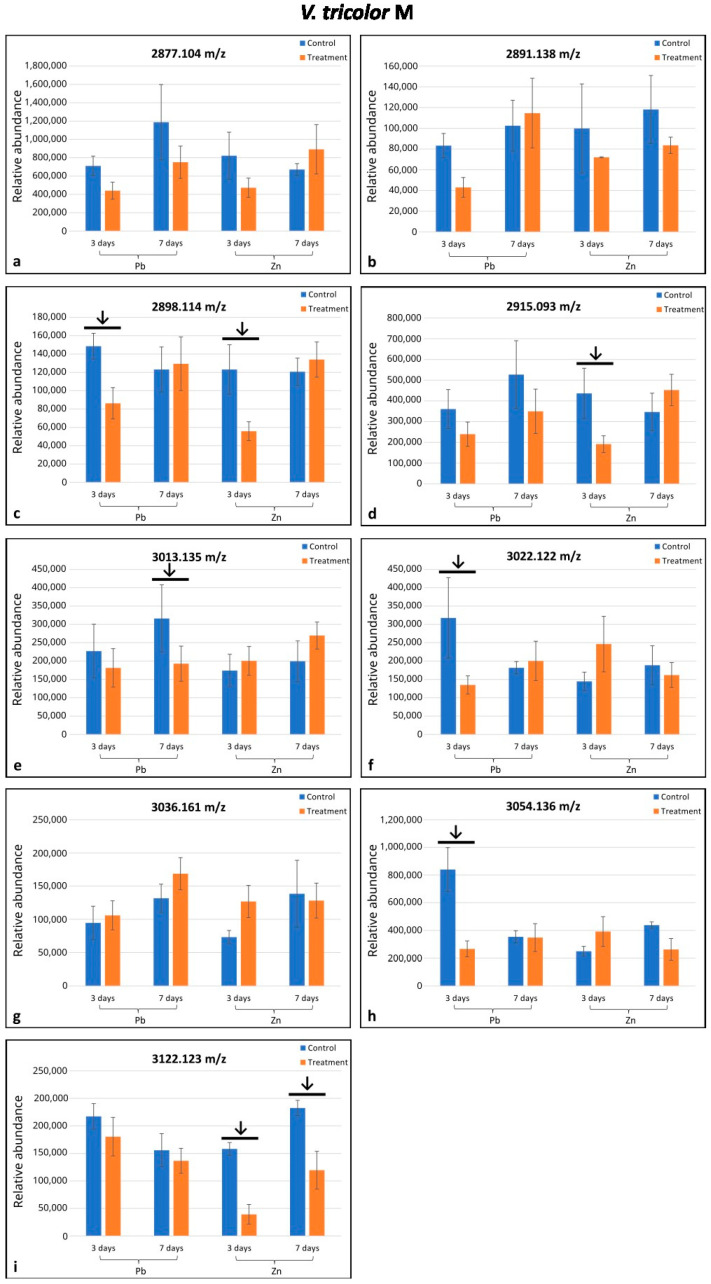
Comparison of mean relative quantities of cyclotides between specimens of *Viola tricolor* from metallicolous (M) population treated with 1000 μM Zn or Pb for three and seven days and non-treated controls. Arrows indicate statistical differences (*p* < 0.05) by Student’ *t*-test. Means and standard deviations were established based on N = 5 replications. Subfigures (**a**–**i**) represent changes in the level of cyclotides of different molecular mass as indicated above each diagram.

**Figure 2:**
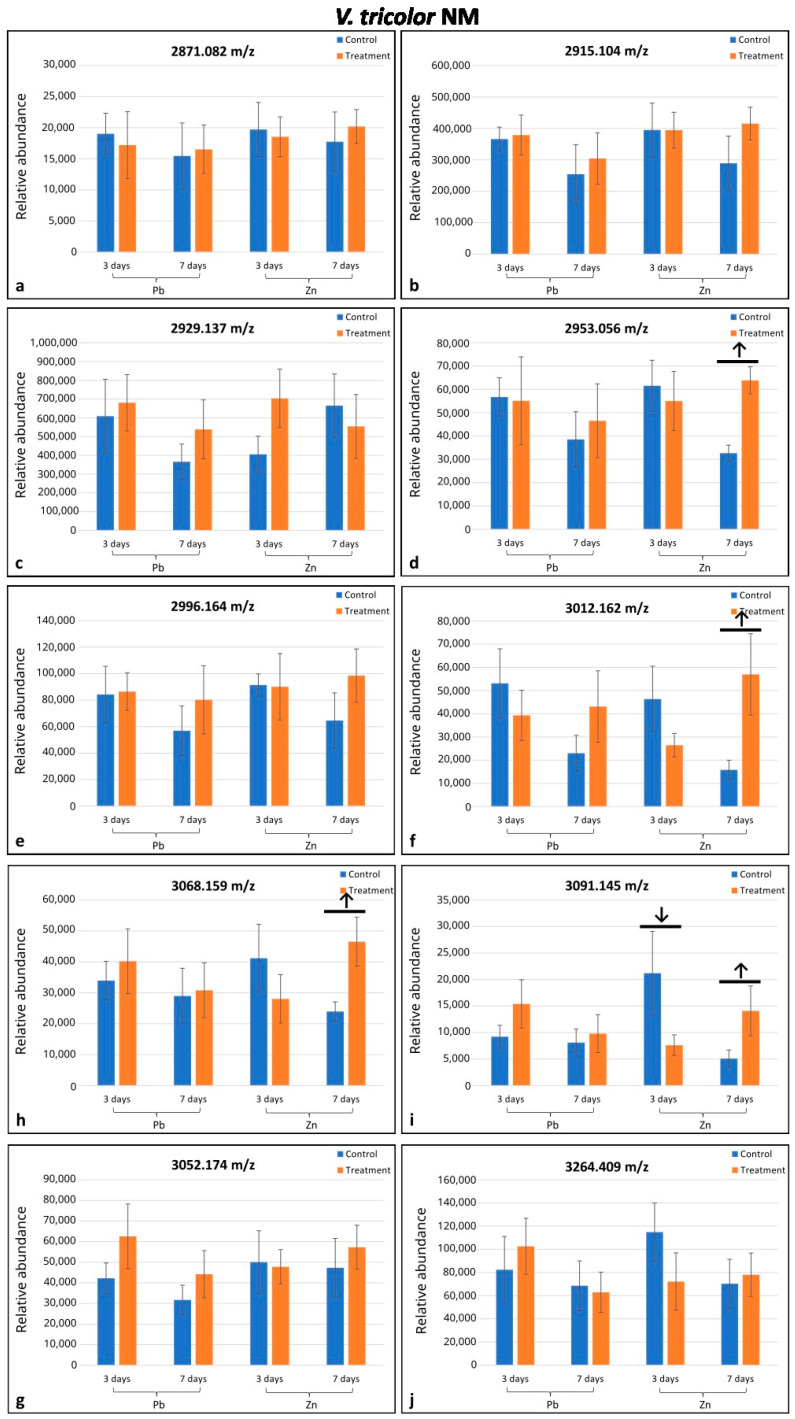
Comparison of mean relative quantities of cyclotides between specimens of *Viola tricolor* from non-metallicolous (NM) population treated with 1000 μM Zn or Pb for three and seven days and non-treated controls. Arrows indicate statistical differences (*p* < 0.05) by Student’ *t*-test. Means and standard deviations established based on N = 5 replications. Subfigures (**a**–**j**) represent changes in the level of cyclotides of different molecular mass as indicated above each diagram.

Altogether, eighteen cyclotides (*m*/*z* range 2871.082–3264.409) were selected from the MALDI-MS analysis; seventeen of them were found in only one of the two populations investigated (eight in M and nine in NM populations), and one was indicated in both populations (Figure 1 and Figure 2). In the M plants, six cyclotides were downregulated (in comparison to the control) in only some treatments depending on the day of treatment and metal type, whereas in NM plants, four cyclotides were upregulated/downregulated in some treatments as indicated by arrows on the histograms (Figure 1c–f,h,i and Figure 2d,f,h,i). Among the nine cyclotides analyzed in the M specimens, four were downregulated in the Pb-treated plants, and three were downregulated in the Zn-treated ones (Figure 1c–f,i). In the NM plants, four cyclotides among the ten detected were upregulated after seven days of Zn treatment and one among those four (*m*/*z* 3091.145) was downregulated after three days of treatment with Zn (Figure 2d,f,h,i). The cyclotide *m*/*z* 2898.114 in M plants was significantly responsive under both Zn and Pb treatment; cyclotides *m*/*z* 3122.123 in M plants and *m*/*z* 3091.409 in NM plants were significantly responsive under different durations of treatment (Figure 1c,i and Figure 2i). Altogether, the seven remaining cyclotides were responsive under only one treatment (Figure 1d–f,h and Figure 2d,f,h).

**Figure 3:**
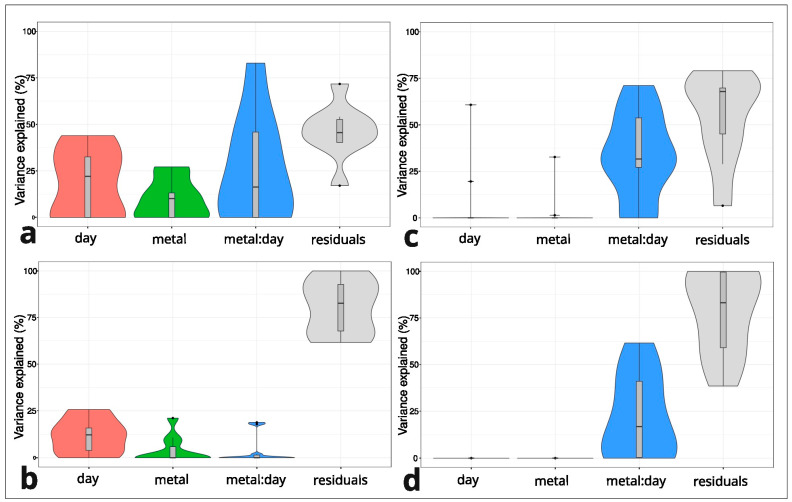
Violin plots representing a comparison of the explained variance in factors (day of treatment and metal dose) in *Viola tricolor* individuals derived from the non-metallicolous (NM) and metallicolous (M) populations treated with 1000 μM Zn or Pb for three and seven days and the non-treated controls. Pb M (**a**), Zn M (**c**), Pb NM (**b**), Zn NM (**d**). Analysis was performed for every single cyclotide.

A linear mixed model was used for every single cyclotide. The day of treatment and dose of metal and their interaction played a crucial role in the explained variance for cyclotides produced by the M individuals but not for the NM plants (Figure 3). The interaction between the day of treatment and dose of metal ranged from 0% to 71% (Zn) and from 0 to 82% (Pb) for the M population (Figure 3a,c). The interaction between the day of treatment and dose of metal was also high (0–61%) in the NM individuals treated with Zn (Figure 3d). In the model produced for cyclotides in NM population treated with Zn, the low percentage of variance explained by the interaction was explained bythe other variables (day of treatment, dose of metal) (Figure 3b). For example, for cyclotide *m*/*z* 3052.173 in the NM individuals with 0% interaction, the influence of the dose of metal was 21%, and the influence of the day of treatment was 15%. The high residual percentage (from 38 to 100%) in Zn treatments of the NM and M specimens indicates that the factors used in the experiment did not explain the model (Figure 3c,d).

## 3. Discussion

In this study, we quantified 18 cyclotides, which is a much lower number than the total number of cyclotides already found in the pseudometallophyte *V. tricolor* [27]. Hellinger et al. [27] identified as many as 164 cyclotides in a study with different goals and methodological approaches, i.e., transcriptome and cyclotide peptidome analyses. Many peaks, likely corresponding to various cyclotides, were observed in the average mass spectra in the present study. However, the selection of cyclotides for further analysis was guided by stringent criteria to ensure robustness and reproducibility. Specifically, we exclusively selected monoisotopic peaks that did not overlap with the isotopic patterns of other cyclotides. This step minimized potential ambiguities in peak identification. Additionally, only cyclotides exhibiting a linear relationship between signal intensity and concentration in extract serial dilutions were included, ensuring that the selected cyclotides represented were quantifiable. The identical methods based on MALDI-MS and the inclusion criteria employed in our earlier studies on different violets, including *V. tricolor*, yielded similar numbers of cyclotides included in the analysis [26,28].

The M and NM specimens share only 1 (*m*/*z* 2915.01) cyclotide among the 18 included in the present analysis. This might be caused by the high phenotypic plasticity of this pseudometallophyte inhabiting different locations or cultivation conditions. Individuals of *V. tricolor* represent extremely high intra- and interpopulational morphological variability; thus, the production of cyclotides may also vary between populations [8,29]. Variability in the number and type of cyclotide between populations may be related to the differences in gene expression or post-translational modifications of cyclotides [30]. Both mechanisms reinforce the range of phenotypic plasticity. In addition, this intraspecies variation in cyclotide production may be influenced by existing high genetic diversity between the M and NM populations of *V. tricolor* [31]. This is emphasized by our findings of the explained variance in factors (day of treatment and metal dose) applied in the current experimental design. Both populations were shown to differ in explained variance. The interaction of factors in the Pb treatment is more meaningful in the M population; meanwhile, in the Zn treatment, this interaction is important in both populations (Figure 3). In conclusion, it seems that both populations, regardless of origin, tolerate high doses of Zn and Pb, but the M population is more sensitive at the level of cyclotide action in response to these metals.

Cyclotides have been shown to have metal binding sites in their molecular structure [25]. A recent study has shown that metals co-localize with cyclotides in vacuoles, thus supporting the hypothesis that plants can use these peptides to bind and detoxify HMs [26]. In the present study, the production of 4/10 (40%) cyclotides in the NM population and 0/9 (0%) cyclotides in the M population increased after HM treatment. On the other hand, 6/9 (67%) cyclotides in the M population and only 1/10 (10%) in the NM population decreased. A similar trend of more relative decreases than increases in cyclotide levels was observed in *V. tricolor* cell suspensions derived from the M population [28]. However, this involved quite different cyclotides than those analyzed in the present study, likely because cell cultures were sustained in growth media supplemented with exogenous plant growth regulators [26]. There are two hypothetical reasons for the decrease in cyclotide abundance under the treatment of HMs. No scientific data currently address cyclotides’ molecular mass or potential denaturation upon binding with heavy metals. However, it can be reasonably predicted that the mass spectrum’s area under the peak would decrease after heavy metal binding, indicating a reduction in detectable cyclotide levels. This could suggest partial denaturation or altered stability of the cyclotide upon metal interaction, compared to its spectrum in the absence of heavy metals. Secondly, under the heavy metal treatment, the plant’s secondary metabolism could be under the cytotoxic effect of HMs, which means that only metabolites crucial for plant survival could be more intensely produced. In such a hypothetical situation, in the case of cyclotides not belonging to the group of compounds of basal metabolism, their decrease in levels seems plausible. Earlier studies have shown that different stress factors, possibly evoking toxic effects, could have resulted in the decreased production of some cyclotides [32,33].

In the current work, the level of cyclotide production in the M specimens under Pb treatment decreases on the third day, which corresponds with a low level of this metal in plant tissues. It changes on the seventh day when cyclotide levels under the Pb treatment return to the level of the control and Pb accumulates substantially (an increase of almost 100 times when comparing the third day with the seventh day) (Table 1). Thus, for the M population, at the beginning of the Pb treatment (third day), the cyclotides could be under cytotoxic effects, whereas at the end of the Pb treatment (seventh day) after recovery, the effects of cytotoxicity are minimalized. It can be hypothesized that the M and NM individuals of *V. tricolor* utilize two different mechanisms involving cyclotides to cope with heavy metal stress. This does not seem surprising as Pb-tolerant and -intolerant genotypes of *A. thaliana* also use different strategies towards HMs [34]. In this species, there are populations with low translocation of Pb and its accumulation into root cell walls and vacuoles or with high translocation of Pb and its efflux to inactive organelles or intracellular spaces. This is associated with the increased expression of certain genes responsible for the thickening of the cell walls, improving Pb accumulation in roots and decreasing its toxicity (tolerant populations of *A. thaliana*), and genes that facilitate the formation of dictyosome vesicles and Pb encapsulation in leaves (intolerant populations) [34]. In turn, in bryophytes adapted to HM-polluted environments, but not in those non-adapted to these environments, γ + β-tocopherol content emerged to be suitable as a plant functional trait biomarker that plays an important role in stress sensing and signaling [35]. In general, acute Cu stress in the laboratory in these mosses led to the downregulation of genes involved in heavy metal tolerance in both the more and the less tolerant populations, but this response was quantitatively higher in the most tolerant one [36]. However, it should be remembered that these studies covered only the level of gene expression, not the content of specific substances, which, after all, could change, if only due to the cytotoxic effects. It is also worth emphasizing that the regulation of heavy metal tolerance is a very complex process involving not only the modulation of gene expression but also such mechanisms as improving the capacity of the antioxidant system, inducing the synthesis of osmoprotectants, the secretion of endogenous hormones, etc. [37]. Babst-Kostecka et al. [38] studied the metallophyte *A. halleri* and found that different populations of the plant behaved differently when it came to dealing with Zn. In populations from lowland, non-metalliferous areas, the plants had higher amounts of Zn in their seeds. This suggests that these plants may have naturally evolved to accumulate more Zn in their tissues. On the other hand, at metalliferous sites, where Zn levels are toxic to plants that cannot tolerate it, plants showed limited movement of Zn into their seeds. This shows how different populations have adapted in different ways to the presence of heavy metals. To date, in-depth comprehensive transcriptomic and metabolomic studies on *V. tricolor* have not been conducted, so there are no other data to compare the mechanisms of tolerance between M and NM populations. However, based on a small number of studies, it is possible to point out at least the following: enhanced callose production in root cell walls acts as a mechanical barrier against HMs in M individuals but not in NM individuals [39]. There is a need for further research on this model species, including comprehensive metabolomic and transcriptomic studies that will allow for the indication of genes and compounds that are involved in the response of M and NM individuals to HMs. Perhaps other detection methods like LC-MS/MS (liquid chromatography with tandem mass spectrometry) would allow comparative studies of the larger pool of cyclotides produced by this species. In conclusion, the present results show that cyclotides produced by the pseudometallophyte *V. tricolor* are involved in its heavy metal tolerance, and the specimens from two distinct (M and NM) populations have developed different strategies and tolerance mechanisms involving cyclotides to mitigate heavy metal stress.

## 4. Materials and Methods

### 4.1. Plant Material

Seeds of *V. tricolor* were collected at two distinct locations. The metallicolous (referred to as M) genotype was acquired from the Bukowno mine heap, located near an operational smelter (N50°16′37″, E19°28′05″). In this area, galena (PbS), calamine (ZnCO_3_, ZnSiO_3_), and sphalerite (ZnS) have been mined since the Middle Ages [40]. The soil parameters—Bukowno in the place of *V. tricolor* growing—are characterized by an average zinc content of 6725 ppm, a lead content of 1769 ppm, and a soil pH of 6.9 [12]. Seeds of the non-metallicolous (referred to as NM) genotype were obtained from the plants grown in the Botanical Garden in Cracow (N50°03′49″, E19°57′19″).

Seeds were sterilized by immersing them in 70% ethanol for 90 s, then in commercial bleach for 12 min, and three times in sterile distilled water for 3, 7, and 10 min, respectively. They were placed on solidified half-strength Murashige and Skoog (MS) medium supplemented with 30 g/L sucrose (all from Sigma-Aldrich, St. Louis, MO, USA), solidified with 8 g/L agar (Duchefa Biochemie, Amsterdam, The Netherlands) at pH 5.7–5.8, and kept in 4 °C for two weeks. Subsequently, the seeds were germinated in a growth chamber at 25 ± 3 °C with a 16 h photoperiod under cool-white fluorescent lamps (light intensity: 70–100 μmol/s/m). Two- to three-week-old seedlings were moved into sterile plastic cups containing the same medium as that used for germination. After 8–10 weeks from germination, and after ensuring that the plants were sterile and free from any fungal, bacterial, or other infections that could alter the cyclotide profile, the plants were transferred to the MS medium containing 0 μM (control) or 1000 μM of Zn(NO_3_)_2_ or Pb(NO_3_)_2_ (65.38 mg/kg of Zn; 207.2 mg/kg of Pb). After 3 and 7 days, 5 individuals per treatment were harvested. Each metal had its own control in separate culture containers. One of two samples obtained by cutting along the length of the plant containing the root, stem, and leaves was allocated for heavy metal measurement analysis, while the second was designated for cyclotide analysis.

### 4.2. Quantification of Content of Heavy Metals in Plants by Atomic Absorption Spectroscopy (AAS)

Dried and weighed samples (5 replicates per treatment) containing the leaves, root, and stem were immersed in 400 μL of a mixture (7:1, *v*:*v*) of ultrapure HNO_3_ and HClO_4_ (both ThermoFisher Scientific, Waltham, MA, USA) and then mineralized for four days at a high temperature starting from 50 °C and increasing up to 150 °C. The resulting digestate was concentrated through evaporation and then diluted using 4 mL of 0.2% HNO_3_. Zn and Pb content was quantified using a Perkin-Elmer AAnalyst 200 (ThermoFisher Scientific, Waltham, MA, USA). To ensure accuracy, three blank samples and two reference samples were analyzed. Zinc was measured in a cuvette using the flame method. Heavy metal concentrations were determined using 1572a pine needles as the standard material [41].

### 4.3. Cyclotide Detection by Matrix Assisted Laser Desorption and Ionization (MALDI-MS)

The MALDI-MS analysis method was performed according to Slazak et al. [33]. Briefly, freeze-dried samples were weighed and placed in separate 2 mL Eppendorf tubes. Then, the samples were extracted using a TissueLyser (Qiagen, Germantown, MD, USA) with 30% acetonitrile (ACN) and 0.1% trifluoroacetic acid (TFA) in MiliQ water using a proportion of 100 µL per 1 mg of plant tissue. To ensure that the concentrations were within the linear response range in MALDI-MS, the extracts were diluted 12-fold. The diluted extracts (0.5 µL of each sample) were spotted on a metal plate, air-dried, and sprayed with 6 passes of MALDI matrix solution (35 mg/mL 2,5-dihydroxybenzoic acid, 50% ACN, and 0.2% TFA) using an automatic matrix sprayer (TM-Sprayer; HTX Technologies, Chapel Hill, NC, USA) with the following parameters: a nitrogen pressure of 6 psi, a solvent flow rate of 70 µL/min, a nozzle head velocity of 110 cm/min, and a 2 mm track spacing. All the spots were analyzed using a MALDI Fourier-transform ion cyclotron resonance (FTICR) (solariX 7T-2ω, Bruker Daltonics, Bremen, Germany) mass spectrometer equipped with a Smartbeam II 2 kHz laser operated in positive ionization mode. The spot areas were imaged at 250 µm lateral resolution, and spectra were obtained by summing up 100 laser shots/pixel in the 300–3500 *m*/*z* range in 2 ω mode with the following parameters: Q1 mass, 500; frequency, 2 MHz; time of flight, 2 ms; and funnel RF amplitude, 150 Vpp. Average mass spectra were generated in FlexImaging, version 4.0 (Bruker Daltonics). The cyclotides in a particular sample were distinguished by their monoisotopic molecular mass between 2.8 and 3.3 kDa. The relative quantitative analysis for monoisotopic cyclotide ions—the mean intensity per pixel per spot (approximately 70 pixels per spot)—was performed in msIQuant software v. 2.0.2.14 [42]. The spectra were normalized against all the data points’ root mean square (RMS). Monoisotopic ions not overlapping with other isotopic pattern peaks in the average spectra were selected. Additionally, serial dilutions of extracts from M and NM samples were also spotted on the MALDI-MS target plate to assess the range of dilutions in which the relation between concentration and intensity is linear for a given ion. Ions showing a linear relation for any of the species and in the linear range in the used extract dilutions were included in the analysis.

### 4.4. Statistical Analyses

Analysis of variance (ANOVA), followed by Tuckey’s test, was used to assess the influence of time and dose of heavy metals on the amount of accumulated heavy metals at *p* ≤ 0.05. The relative quantity of cyclotides in a few samples was analyzed using Student’ *t*-test (control and treatment at each time). To understand the drivers of variation in the production of each cyclotide, variance partitioning was performed using the variancePartition package. variancePartition uses a linear mixed model to partition the variance attributable to multiple variables in the data. Mixed-model analysis of variance is necessary to quantify the biological and technical components of the experimental variance as well as their interactions and to split the total variance into between-subject and within-subject components [43]. Analyses were performed using R Project for Statistical Computing (v.4.4.2.) and Microsoft Excel 16.

## Figures and Tables

**Table 1:** The mean Zn or Pb concentrations [mg kg^−1^] and bioaccumulation factors +/− [standard deviation] in *Viola tricolor* metallicolous (M) and non-metallicolous (NM) specimens treated with 1000 μM for 3 and 7 days. Asterisks indicate statistically significant differences in heavy metal concentrations/bioaccumulation factor in the interaction of population and time (*p* ≤ 0.05). Means and standard deviations were established based on N = 5 replications.

		Heavy Metal Concentration (mg kg^−1^)		Bioaccumulation Factor	
Population Days of Treatment		Pb	Zn	Pb	Zn
M	3	2.835 ± [0.938] *	284.206 ± [110.516]	0.013 ± [0.004] *	4.372 ± [1.700]
	7	277.360 ± [108.281]	601.721 ± [197.362]	1.339 ± [0.523]	9.257 ± [3.036]
NM	3	155.818 ± [58.976]	443.771 ± [139.720]	0.752 ± [0.284]	6.827 ± [2.149]
	7	199.207 ± [54.611]	705.695 ± [358.215]	0.962 ± [0.263]	10.856 ± [5.511]

## Data Availability

The data presented in this study are available on request from the corresponding authors.
